# Supplementary material for: The impact of hospital pharmacists’ psychological contracts on medication adherence management: The mediating role of job burnout
Source: PLoS One. 2026 Apr 29;21(4):e0347880. doi: 10.1371/journal.pone.0347880 (PMC13127971; doi:10.1371/journal.pone.0347880)
Supplement: S1 File — (DOCX) [file pone.0347880.s001.docx]

**supplementary material—Expert Survey Results.**

| **Subscale** | **Key Indicators** | **Mean** | **Full Score Frequency** | **CV** | **Expert Comments** |  |
| --- | --- | --- | --- | --- | --- | --- |
| Pharmacist's attitude towards participating in patient medication adherence management | #1. Participating in patient medication adherence management is a requirement of pharmacists' professional ethics. | 4.63 | 62.50% | 0.10 | Modified to "professional ethics" to "responsibility" |  |
|  | 2. Participating in patient medication adherence management is not necessarily part of pharmacists' responsibilities. | 4.19 | 43.75% | 0.24 |  |  |
|  | #3. Pharmacists play a leading role in improving patient medication adherence. | 4.31 | 31.25% | 0.11 | Modified to "Pharmacists should play a leading role in patient medication adherence management." |  |
|  | 4. Pharmacists' management of patient medication adherence plays a crucial role in treatment outcomes. | 4.38 | 43.75% | 0.14 |  |  |
|  | 5. Participating in medication adherence management is one way for pharmacists to achieve self-worth. | 4.56 | 68.75% | 0.15 |  |  |
|  | 6. Pharmacists should regularly assess patients' medication adherence. | 4.69 | 75.00% | 0.12 |  |  |
|  | 7. Pharmacists should regularly educate patients on medication adherence. | 4.81 | 81.25% | 0.08 |  |  |
|  | 8. Pharmacists should actively research and use various tools and methods to improve patient medication adherence. | 4.63 | 68.75% | 0.13 |  |  |
|  | *9. Whether patients adhere to medication is their own choice and is the responsibility of the patient or prescribing physician, not the pharmacist. | 2.75 | 25.00% | 0.60 | Delete, as it duplicates other items. |  |
| The behaviors of pharmacists in participating in patient medication adherence management | 1. Establish patient health records, document medication information, and implement personalized adherence management. | 4.69 | 75.00% | 0.12 |  | |
|  | #2. **Monitor patients' medication adherence through methods such as pill counting and blood drug concentration monitoring.** | 4.44 | 50.00% | 0.14 | Modified to "Monitoring patient medication adherence through inquiry or monitoring methods (such as pill count, blood drug concentration monitoring, and clinical indicator testing, etc.)" | |
|  | #3. Assess the psychological and pathological factors in patients that may affect medication adherence. | 4.63 | 62.50% | 0.10 | Modified to “Assess psychological and pathophysiological factors (such as advanced age, memory decline, and decreased mobility) and social factors (such as previous treatment failures, medication accessibility, and financial burden related to medication use) that may affect the patient's medication adherence.” | |
|  | #4. Regularly inquire about objective factors that may affect patients' adherence to prescribed medications (such as side effects of the medication, financial burden related to medication use, complexity of the medication regimen, etc.). | 4.69 | 75.00% | 0.12 | Remove "financial burden related to medication use" | |
|  | #5. Verify the medication usage of patients with chronic diseases, especially the use of medication devices. | 4.56 | 56.35% | 0.11 | Modified to “Check the mastery of medication usage methods among patients with chronic diseases and implement necessary educational measures as needed.” | |
|  | 6. Assess the patient's medication adherence using compliance evaluation scales (such as MMAS, TAI, etc.) based on the patient's specific conditions. | 4.63 | 62.50% | 0.10 |  | |
|  | #7. Conduct medication adherence education activities through patient education lectures and community outreach. | 4.50 | 56.25% | 0.14 | Modified to "Conduct medication adherence education activities through patient education lectures, community outreach, and playing medication education videos." | |
|  | #8. Correct the patient's misconception of stopping medication as soon as their condition shows slight improvement. | 4.75 | 75.00% | 0.09 | Modified to "correct the perception and behavior of patients discontinuing medication on their own, emphasizing the importance of regular medical visits and the dangers of arbitrarily stopping medication." | |
|  | #9. Implement standardized communication regarding medication therapy, covering the nature of the disease, medication options, treatment expectations, and more. | 4.50 | 56.25% | 0.14 | Modified to "Standardize communication regarding medication therapy, covering the nature of the disease, medication options, treatment expectations, safety, and cost-effectiveness." | |
|  | #10. Promote medication adherence-related communication among patients. | 3.75 | 31.25% | 0.35 | Modify to "Regularly organize group discussions among patients to exchange experiences on overcoming medication barriers and coping with drug side effects." | |
|  | 11. The pharmacist regularly communicates with the patient and their family members or caregivers to ensure that the patient is supervised in taking their medication according to the prescribed regimen. | 4.63 | 62.50% | 0.10 |  | |
|  | #12. Collaborate with the medical team to provide feedback on patient adherence and offer recommendations for medication selection. | 4.69 | 68.75% | 0.10 | Change"offer recommendations for medication selection." to "provide recommendations for adjustments to drug treatment plans." | |
|  | 13. **Provide patients with health consultations and guidance related to rational medication use, including diet, exercise, and stress management, and offer personalized professional services for special patient groups.** | 4.38 | 43.75% | 0.14 |  | |
|  | #14. During the patient consultation process, clarify and verify the medication instructions. | 4.44 | 56.25% | 0.16 | Modify to “During the medication guidance process, promptly correct any inappropriate medication behaviors of the patient, demonstrate the correct method, and ensure that they understand and master the proper way to take the medication.” | |
|  | *15. Make clear notes on the medications taken by patients who are forgetful or elderly. | 4.69 | 68.75% | 0.10 | Delete, as it duplicates other items. | |
|  | 16. Encourage patients to develop self-medication monitoring and cultivate awareness of self-adherence management. | 4.69 | 75.00% | 0.12 |  | |
|  | 17. Using the Health Belief Model to enhance patients' perception of their susceptibility to illness and the severity of the disease. | 4.38 | 43.75% | 0.14 |  | |
|  | 18. Plan, monitor, and evaluate the medication regimen, and establish a treatment plan that is tailored to the patient’s preferences. | 4.38 | 43.75% | 0.14 |  | |
|  | #19. Use medication cards or instructional leaflets to remind and guide patients to take their medications correctly. | 4.38 | 43.75% | 0.14 | Modify to "Use drug cards or medication instruction leaflets to remind and guide patients to take their medications correctly, and make clear notes on the medications taken by forgetful or elderly patients." | |
|  | #20. Guide patients to use self-service medical devices (such as blood pressure monitors and glucometers) properly to promote adherence to prescribed medication regimens. | 4.31 | 31.25% | 0.11 | Modify to“Guide patients in the correct use of self-service medical devices (such as blood pressure monitors and glucometers) and instruct them on how to respond to abnormal monitoring results.” | |
|  | 21. Promote the use of smart pill boxes for patients who are eligible (features include real-time reminders and real-time synchronization of medication records, etc.). | 4.13 | 37.50% | 0.19 |  | |
|  | #22. Patients are regularly reminded of medication use and drug replenishment through various communication means, including phone calls, text messages, pharmaceutical service platforms, QQ, WeChat, and others. | 4.38 | 56.25% | 0.18 | Modify to “Regularly remind patients of medication use, replenish medications, and provide professional answers to medication-related questions through communication means such as telephone, text messages, pharmaceutical service platforms, QQ, and WeChat.” | |
|  | *23. Research or use of artificial intelligence (AI) in managing patients' medication. | 3.69 | 12.50% | 0.25 | Delete, as it duplicates other items. | |
|  | #24. Keep abreast of the latest information and developments in first-line medications both domestically and internationally, and actively utilize new theories, knowledge, and technologies in the field of pharmaceutical and medical sciences to guide patients in the rational use of medications. | 4.50 | 56.25% | 0.14 | Modify to “Keep abreast of the latest information and development trends of first-line medications both domestically and internationally, and actively utilize new theories, knowledge, and technologies in the field of pharmaceutical medicine to guide rational medication use in clinical practice and for patients.” | |
| Add new entry：7. Verify the medication information of transferred patients, coordinate the medication plans, and ensure the continuity of medical care; 8. Regularly assess the patient's acceptance and implementation of personalized medication guidance or adherence reminders; 15. Based on the patient's feedback on drug efficacy or adverse reactions, promptly optimize medication guidance and intervention measures. | | | | | | |

*indicates that the item was removed; #indicates that the item was modifi
