# Supplementary material for: The impact of hospital pharmacists’ psychological contracts on medication adherence management: The mediating role of job burnout
Source: PLoS One. 2026 Apr 29;21(4):e0347880. doi: 10.1371/journal.pone.0347880 (PMC13127971; doi:10.1371/journal.pone.0347880)
Supplement: Attachment — Submitted filename: Response to Reviewers.docx [file pone.0347880.s003.docx]

***Fushan Tang, Professor, Ph.D***

Department of Clinical Pharmacy, Key Laboratory of Basic Pharmacology of Guizhou Province and School of Pharmacy, Zunyi Medical University, Zunyi 563006, China.

Email：fstang@vip.163.com; fstang@zmc.edu.cn.

*Tel*: *86851 28642337, Fax: 86851 28642334*

1 September 2025

Dear Editors,

Thank you very much for your letter dated July 23, 2025, informing us about the revisions required for our manuscript titled “The impact of hospital pharmacists' psychological contracts on medication adherence management: the mediating role of job burnout” (Manuscript ID: PONE-D-25-16578). We sincerely appreciate the valuable guidance, comments, and suggestions provided by the reviewers.

We have carefully reviewed all the comments and revised the manuscript accordingly. All modifications have been highlighted in the manuscript for easy identification. Additionally, we have provided detailed point-by-point responses to the reviewers' comments below.

**Response to Journal Requirements：**

Comments 1: Please ensure that your manuscript meets PLOS ONE's style requirements, including those for file naming. The PLOS ONE style templates can be found at

https://journals.plos.org/plosone/s/file?id=wjVg/PLOSOne_formatting_sample_main_body.pdf and

[https://journals.plos.org/plosone/s/file?id=ba62/PLOSOne_formatting_sample_title_authors_affiliations.pdf](https://track.editorialmanager.com/CL0/https:%2F%2Fjournals.plos.org%2Fplosone%2Fs%2Ffile%3Fid=ba62%2FPLOSOne_formatting_sample_title_authors_affiliations.pdf/1/010f01983775f6c3-a579771b-f00d-4b8a-a21a-32604c42eb54-000000/fRNssJQSfAlCXVVu6RST0qjJIaeDevU8ZtB9-l81E6s=220)

Response 1: Thank you for your reminder. We have carefully reviewed the PLOS ONE style requirements and formatting guidelines, including file naming conventions, and have revised our manuscript accordingly to ensure full compliance with the journal’s standards.

Comments 2: Please state what role the funders took in the study. If the funders had no role, please state: "The funders had no role in study design, data collection and analysis, decision to publish, or preparation of the manuscript." If this statement is not correct you must amend it as needed. Please include this amended Role of Funder statement in your cover letter; we will change the online submission form on your behalf.

Response 2: Thank you for your comment. We confirm that the funders had no role in study design, data collection and analysis, decision to publish, or preparation of the manuscript. They provided financial support only. We have added this amended Role of Funder statement to the cover letter as requested.

Comments 3: Your ethics statement should only appear in the Methods section of your manuscript. If your ethics statement is written in any section besides the Methods, please move it to the Methods section and delete it from any other section. Please ensure that your ethics statement is included in your manuscript, as the ethics statement entered into the online submission form will not be published alongside your manuscript.

Response 3: Thank you for your comment. We have ensured that the ethics statement appears only in the Methods section of the manuscript and have removed it from any other sections. The statement is now fully compliant with PLOS ONE’s requirements.

Comments 4: In the online submission form, you indicated that the datasets used and analyzed in this study can be obtained from the corresponding author upon reasonable request. All PLOS journals now require all data underlying the findings described in their manuscript to be freely available to other researchers, either a. In a public repository, b. Within the manuscript itself, or c. Uploaded as supplementary information. This policy applies to all data except where public deposition would breach compliance with the protocol approved by your research ethics board. If your data cannot be made publicly available for ethical or legal reasons (e.g., public availability would compromise patient privacy), please explain your reasons on resubmission and your exemption request will be escalated for approval.

Response 4: Relevant data supporting the findings of this study can be made available upon reasonable request. In addition to contacting the corresponding author Professor Fushan Tang (Email: fstang@zmu.edu.cn), data access requests may also be directed to Deputy Chief Pharmacist Chengchen Yin at Renhuai Traditional Chinese Medicine Hospital (Email: 824196010@qq.com), who serves as an institutional representative not listed as an author. To ensure persistent and long-term availability, all study data are securely stored in two independent locations: the Clinical Pharmacy Office of Zunyi Medical University and Renhuai Traditional Chinese Medicine Hospital.

**Response to the Reviewers’ Comments:**

**Reviewer 1**

Comments 1: In the Introduction, it is not clear on what basis the correlation between the variables Psychological Contracts, Medication Adherence and Job Burnout was measured. Was this based on a model or theory or was it based on a literature review?

Response 1: Thank you for your valuable comment. We agree that the theoretical basis for the proposed relationships needed to be clarified. In the revised Introduction, we have added a paragraph to explain that our research framework is grounded in psychological contract theory and social exchange theory. We highlight that fulfillment of psychological contracts enhances pharmacists’ motivation and professional engagement, whereas breaches may foster job burnout, which in turn undermines pharmacists’ behaviors in supporting patients’ medication adherence. These links are supported by prior studies on healthcare professionals, providing a sound theoretical and empirical basis for our research model. Please see lines 59–66 of the revised manuscript.

Comments 2: The method is ambiguous. For example, it is written that A comprehensive literature search was conducted to prepare a draft scale "Pharmacist's Attitudes and Behaviors Toward Patient Medication Adherence Management". The literature search was also a systematic review study. However, there is no information about the method of this systematic review study. For example, the number of articles reviewed, the number of articles excluded, the eligibility of articles, etc. It would have been better to add a prism diagram.

Response 2: Thank you for your insightful comment. We acknowledge that our description may have caused confusion. Our literature search was intended as an exploratory review to inform the development of the scale, rather than a formal systematic review. Therefore, we did not apply PRISMA methodology or report article inclusion/exclusion numbers. To avoid misunderstanding, we have revised the Methods section to clarify that the literature search was exploratory in nature and primarily aimed to generate an item pool, which was then refined through expert consultations.

Comments 3: How was the item pool formed in the preparation of this scale and how many items did it have? What steps were taken in the next step, Delphi Expert Consultation?

Response 3: Thank you very much for your insightful comments. Regarding your question:

(1) Formation of the item pool

The item pool was developed through a targeted literature review rather than a formal systematic review. We searched both Chinese (CNKI, Wanfang, VIP) and international databases (PubMed, Web of Science) using keywords such as "Medication Adherence," "Pharmacist," "Psychological Contract," and "Management." Relevant literature, policy documents, and practice guidelines were synthesized to identify common domains of pharmacists’ attitudes and behaviors related to patient medication adherence management. Based on this process, an initial item pool was generated, including 9 attitude items and 24 behavior items, for a total of 33 items.

(2) Delphi expert consultation

The Delphi process was conducted in a single round with 16 experts. Experts rated the importance and clarity of each item and provided qualitative feedback. Based on their suggestions, redundant or ambiguous items were revised or deleted, and consensus was reached on the final set of items. This process ensured content validity and expert agreement regarding the scale structure.

Comments 4: Expert Evaluation and Data Analysis was based on a formula. What is the reference of this formula? And how is it evaluated? In what range is accepted?

Response 4: Thank you for your valuable comment. The formula used for calculating the expert authority coefficient (Cr) is based on standard methodology commonly applied in Delphi studies in health services research. Specifically, Cr is calculated as (Cs + Ca)/2, where Cs represents the expert’s familiarity with the questionnaire and Ca reflects the basis of the expert’s judgment. This approach has been adopted in previous studies (Dai F, Wei K, Chen Y, Ju M. Construction of an index system for qualitative evaluation of undergraduate nursing students innovative ability: A Delphi study. J Clin Nurs. 2019;28(23-24):4379-4388. doi:10.1111/jocn.15020).

In our study, a Cr value ≥ 0.70 was considered to indicate good reliability, consistent with the thresholds reported in earlier methodological research (Hu Q, Qin Z, Zhan M, Wu B, Chen Z, Xu T. Development of a trigger tool for the detection of adverse drug events in Chinese geriatric inpatients using the Delphi method. Int J Clin Pharm. 2019;41(5):1174-1183. doi:10.1007/s11096-019-00871-x). The ranges for Cs (0.1–0.9) and Ca (0.1–0.5, depending on the strength of the judgment basis) are presented in Table 1 of our manuscript.

Regarding evaluation, expert authority was judged as acceptable when Cr ≥ 0.70, expert opinion concentration was confirmed by an average importance score ≥ 3.5, and coordination of expert opinions was verified with CV < 0.30 and Kendall’s W showing P < 0.05, following established Delphi methodology (de Goumoëns V, Lefrançois LE, Forestier A, et al. Bachelor nursing competencies to care for children in hospital and home settings: A Delphi study. Nurse Educ Today. 2025;145:106487. doi:10.1016/j.nedt.2024.106487).

Comments 5: In the Participant Recruitment stage, what was the sample size and sampling method? How was the validity of the scale determined?

Response 5: We appreciate the reviewer’s insightful comment. In the Participant Recruitment stage, a stratified random cluster sampling method was adopted to recruit participants from tertiary, secondary, and primary healthcare institutions in Zunyi and Bijie, China. Between November and December 2024, a total of 196 valid responses were obtained.

Regarding scale validity, the Kaiser–Meyer–Olkin (KMO) measure and Bartlett’s test of sphericity were employed to evaluate sampling adequacy and the suitability of the data for factor analysis. A KMO value above 0.7 together with a significant Bartlett’s test result (P < 0.05) confirmed that the data were appropriate for further analysis, thereby supporting the structural validity of the scale in line with the theoretical framework.

Comments 6: On what basis was the Expert's judgment made?

Response 6: The experts’ judgments were based on their professional background and extensive experience in hospital pharmacy and clinical practice. All invited experts held senior professional titles (associate chief pharmacist or chief pharmacist) and had more than ten years of work experience. They evaluated each item in terms of its importance, clarity, and relevance to pharmacists’ involvement in patient medication adherence management, and provided qualitative feedback. This ensured that the scale items reflected both theoretical rigor and practical applicability from the perspective of frontline pharmacists.

Comments 7: The tables provided are too many. 12 tables!

Response 7: We sincerely appreciate the reviewer’s suggestion regarding the number of tables. We fully understand the concern; however, the tables included in the manuscript serve distinct and essential purposes. Specifically, they present (1) the Delphi process results, (2) the reliability and validity testing of the scale, (3) the analysis of the relationship between pharmacists’ psychological contract and medication adherence management, and (4) the mediation analysis of job burnout. Each table corresponds to a separate stage of data processing or statistical analysis, and merging or removing them may compromise the clarity and completeness of the findings. For this reason, we respectfully maintain all tables to ensure transparency and scientific rigor in presenting the study results.

Comments 8: The discussion, considering the results of other studies, needs a general review.

Response 8: Thank you for your valuable comment. In response, we have thoroughly revised the Discussion section to provide a more comprehensive and balanced review of the literature. Specifically, we have incorporated comparative analyses, highlighting both consistencies with previous studies and observed discrepancies, along with potential explanations. We have emphasized the novelty of our study, particularly how it addresses gaps in the literature regarding hospital pharmacists’ roles in medication adherence management and the mediating effect of burnout. Additionally, the logical structure of the Discussion has been strengthened, following a clear sequence: major findings, consistencies with prior research, observed differences and possible explanations, study significance and innovations, and limitations with directions for future research. These revisions ensure that the Discussion is both comprehensive and well contextualized within the current body of evidence. Please see lines 214–271 of the revised manuscript.

**Reviewer 2**

Comments 1: (line 99) in Method section related to detail on 'participant recruitment', please mention if any incentives were given or not.

Response 1: We appreciate the reviewer’s valuable comment. We have now clarified in the Methods – Participant recruitment section that no financial or material incentives were provided for participation. The revised sentence reads: “*No financial or material incentives were provided for participation*”.

Comments 2: (line 126) in Results, in table 2, add explanation to 'research field' showing 2 participants as 'others', which were these?

Response 2: Thank you for your careful observation. In our questionnaire, the category “*Other*” was predefined to capture participants whose research fields did not fit within *Clinical Pharmacy*, *Hospital Pharmacy*, or *Pharmaceutical Management*. The two experts classified under “*Other*” were engaged in areas such as Pharmaceutical Analysis, Pharmaceutics, and other pharmacy-related disciplines. Although these fields play important roles within hospital pharmacy practice, their direct contribution to pharmaceutical management research is comparatively limited, which is why they were categorized as “*Other*.” This approach ensured inclusivity and avoided misclassification of participants’ expertise. We have now clarified this point in the Results section (Table 2 note: Other: fields such as Pharmaceutical Analysis and Pharmaceutics).

Comments 3: (line 144) in table 5, please mention the unit of currency for 'income level'.

Response 3: Thank you for your careful observation. The income levels reported in Table 5 are expressed in Chinese Yuan (CNY, ¥). We have added this clarification to the table note.

Comments 4: (line 239) in discussion, add that burnout screening should be done quarter-wise or biannually, for surveillance or quality measure, to identify areas of improvement through targeted quality improvement initiatives.

Response 4: Thank you for your valuable suggestion. We agree that regular burnout screening is essential for early detection and continuous quality improvement. Accordingly, we have revised the discussion to include the recommendation that hospitals conduct burnout screening on a quarterly or biannual basis as part of surveillance and quality measurement strategies, enabling timely interventions and targeted improvements (see Discussion, lines 262–271).

We sincerely appreciate the constructive feedbacks from the editor and reviewers, which have greatly helped us improve our manuscript. We hope that the revised version meets the journal’s standards and look forward to your kind evaluation.

Best regards,

Fushan Tang
