# Supplementary material for: The impact of hospital pharmacists’ psychological contracts on medication adherence management: The mediating role of job burnout
Source: PLoS One. 2026 Apr 29;21(4):e0347880. doi: 10.1371/journal.pone.0347880 (PMC13127971; doi:10.1371/journal.pone.0347880)
Supplement: Attachment — Submitted filename: Response to Reviewers-R2.docx [file pone.0347880.s004.docx]

***Fushan Tang, Professor, Ph.D***

Department of Clinical Pharmacy, Key Laboratory of Basic Pharmacology of Guizhou Province and School of Pharmacy, Zunyi Medical University, Zunyi 563006, China.

Email：fstang@vip.163.com; fstang@zmc.edu.cn.

*Tel*: *86851 28642337, Fax: 86851 28642334*

10 January 2026

Dear Editors,

Thank you very much for your letter dated December 8, 2025, informing us about the revisions required for our manuscript titled “The impact of hospital pharmacists' psychological contracts on medication adherence management: the mediating role of job burnout” (Manuscript ID: PONE-D-25-16578). We sincerely appreciate the valuable guidance, comments, and suggestions provided by the reviewers.

We have carefully reviewed all the comments and revised the manuscript accordingly. All modifications have been highlighted in the manuscript for easy identification. Additionally, we have provided detailed point-by-point responses to the reviewers' comments below.

**Reviewer 1**

Comments 1: In the Introduction, it is not clear on what basis the correlation between the variables Psychological Contracts, Medication Adherence and Job Burnout was measured. Was this based on a model or theory or was it based on a literature review?

Response 1: Thank you for your valuable comments. The proposed relationships among psychological contract, medication adherence management, and job burnout in this study are not based on exploratory analyses or ad hoc assumptions, but are grounded in established theoretical frameworks and supported by existing literature.

Specifically, this study is primarily informed by social exchange theory and conservation of resources theory (Dee J, Dhuhaibawi N, Hayden JC. Int J Clin Pharm. 2023;45(5):1027–1036). According to social exchange theory, employees’ perceptions of psychological contract fulfillment or breach shape their evaluations of organizational support and reciprocal obligations. Conservation of resources theory further posits that psychological contract breaches lead to ongoing depletion of individuals’ psychological resources, thereby increasing job burnout. Prior research has consistently identified job burnout as a key mediating mechanism through which psychological contracts influence work attitudes and outcomes.

In addition, existing literature indicates that healthcare professionals’ work engagement and well-being are closely associated with their performance in patient-centered professional behaviors, including medication adherence management (Ring M, Hult M. J Adv Nurs. 2025;81(3):1323–1331). Fulfillment of the psychological contract enhances healthcare professionals’ motivation and sense of responsibility, whereas elevated burnout may undermine their engagement in patient management and service delivery.

Therefore, the hypothesized relationships among the study variables were developed under these theoretical frameworks and in alignment with prior empirical evidence, providing a clear theoretical and literature-based foundation for the study. Please see lines 62–70 of the revised manuscript.

Comments 2: The method is ambiguous. For example, it is written that A comprehensive literature search was conducted to prepare a draft scale "Pharmacist's Attitudes and Behaviors Toward Patient Medication Adherence Management". The literature search was also a systematic review study. However, there is no information about the method of this systematic review study. For example, the number of articles reviewed, the number of articles excluded, the eligibility of articles, etc. It would have been better to add a prism diagram.

Response 2: Thank you for your insightful comments. We acknowledge that the description of the literature review may have caused confusion. The purpose of the literature search in this study was to provide an exploratory review to support scale development, rather than to conduct a formal systematic review. Accordingly, PRISMA methodology was not applied, and the numbers of included and excluded articles were not reported.

To avoid misunderstanding, we have clarified this point in the Methods section by explicitly stating: “The literature review had an exploratory nature, with the primary aim of developing an initial item pool, which was subsequently refined and optimized through expert consultation” (lines 94–95).

Comments 3: How was the item pool formed in the preparation of this scale and how many items did it have? What steps were taken in the next step, Delphi Expert Consultation?

Response 3: Thank you very much for your insightful comments. Regarding your question:

(1) Formation of the item pool

The item pool was developed through a targeted literature review rather than a formal systematic review. We searched both Chinese (CNKI, Wanfang, VIP) and international databases (PubMed, Web of Science) using keywords such as "Medication Adherence," "Pharmacist," "Psychological Contract," and "Management." Relevant literature, policy documents, and practice guidelines were synthesized to identify common domains of pharmacists’attitudes and behaviors related to patient medication adherence management. Based on this process, an initial item pool was generated, including 9 attitude items and 24 behavior items, for a total of 33 items.

(2) Delphi expert consultation

The Delphi process was conducted in a single round with 16 experts. Experts rated the importance and clarity of each item and provided qualitative feedback. Based on their suggestions, redundant or ambiguous items were revised or deleted, and consensus was reached on the final set of items. This process ensured content validity and expert agreement regarding the scale structure.

Comments 4: Expert Evaluation and Data Analysis was based on a formula. What is the reference of this formula? And how is it evaluated? In what range is accepted?

Response 4: Thank you for your valuable comment. The formula used for calculating the expert authority coefficient (Cr) is based on standard methodology commonly applied in Delphi studies in health services research. Specifically, Cr is calculated as (Cs + Ca)/2, where Cs represents the expert’s familiarity with the questionnaire and Ca reflects the basis of the expert’s judgment. This approach has been adopted in previous studies (Dai F, Wei K, Chen Y, Ju M. Construction of an index system for qualitative evaluation of undergraduate nursing students innovative ability: A Delphi study. J Clin Nurs. 2019;28(23-24):4379-4388. doi:10.1111/jocn.15020).

In our study, a Cr value ≥ 0.70 was considered to indicate good reliability, consistent with the thresholds reported in earlier methodological research (Hu Q, Qin Z, Zhan M, Wu B, Chen Z, Xu T. Development of a trigger tool for the detection of adverse drug events in Chinese geriatric inpatients using the Delphi method. Int J Clin Pharm. 2019;41(5):1174-1183. doi:10.1007/s11096-019-00871-x). The ranges for Cs (0.1–0.9) and Ca (0.1–0.5, depending on the strength of the judgment basis) are presented in Table 1 of our manuscript.

Regarding evaluation, expert authority was judged as acceptable when Cr ≥ 0.70, expert opinion concentration was confirmed by an average importance score ≥ 3.5, and coordination of expert opinions was verified with CV < 0.30 and Kendall’s W showing P < 0.05, following established Delphi methodology (de Goumoëns V, Lefrançois LE, Forestier A, et al. Bachelor nursing competencies to care for children in hospital and home settings: A Delphi study. Nurse Educ Today. 2025;145:106487. doi:10.1016/j.nedt.2024.106487).

Comments 5: In the Participant Recruitment stage, what was the sample size and sampling method? How was the validity of the scale determined? On what basis was the Expert's judgment made?

Response 5: We appreciate your insightful comments. During participant recruitment, a stratified random cluster sampling method was employed to recruit participants from tertiary, secondary, and primary healthcare institutions in the Zunyi and Bijie regions of China. A total of 196 valid questionnaires were collected between November and December 2024.

Regarding scale validity, sampling adequacy and the suitability of the data for factor analysis were assessed using the Kaiser–Meyer–Olkin (KMO) measure and Bartlett’s test of sphericity. KMO values greater than 0.7 and statistically significant Bartlett’s test results (P < 0.05) indicated that the data were appropriate for factor analysis, thereby supporting the construct validity of the scale in accordance with its theoretical framework.

Expert judgment was primarily based on both the experts’ judgment criteria and quantitative evaluation results. Specifically, experts’ judgment criteria included practical experience, theoretical analysis, relevant literature, and personal judgment. These criteria were weighted according to established methods in prior studies and were used to calculate the expert authority coefficient (Cr). In addition, experts rated the importance of each item using a 5-point Likert scale. The mean importance score, coefficient of variation (CV), and Kendall’s coefficient of concordance (W) were jointly used to assess the concentration and consistency of expert opinions. Ultimately, decisions regarding item retention were made by integrating quantitative indicators with experts’ qualitative feedback.

Comments 6: The tables provided are too many. 12 tables!

Response 6: We sincerely appreciate the your suggestion regarding the number of tables. We fully understand the concern; however, the tables included in the manuscript serve distinct and essential purposes. Specifically, they present (1) the Delphi process results, (2) the reliability and validity testing of the scale, (3) the analysis of the relationship between pharmacists’ psychological contract and medication adherence management, and (4) the mediation analysis of job burnout. Each table corresponds to a separate stage of data processing or statistical analysis, and merging or removing them may compromise the clarity and completeness of the findings. For this reason, we respectfully maintain all tables to ensure transparency and scientific rigor in presenting the study results.

Comments 7: The discussion, considering the results of other studies, needs a general review.

Response 7: We thank for your additional valuable comments. In response to the suggestion that the Discussion should provide a more comprehensive synthesis of relevant studies, we have systematically restructured and substantially expanded the Discussion section in the previous round of revisions.

Specifically, the revised Discussion now provides an integrated evaluation of our findings in relation to existing literature from the following perspectives:

(1) Drawing on prior studies, we systematically discuss the role of pharmacists in medication adherence management and compare our findings with those of related research, highlighting both consistencies and discrepancies;

(2) Guided by psychological contract theory and existing empirical evidence, we further analyze the relationship between psychological contract fulfillment and pharmacists’ attitudes and behaviors, and extend this discussion to the context of medication adherence management;

(3) With reference to relevant studies, we examine the mediating role of job burnout in the relationship between psychological contract and engagement in adherence management, and discuss potential underlying mechanisms;

(4) Finally, the Discussion concludes by outlining organizational-level practical implications and directions for future research, informed by the existing literature.

Together, these revisions constitute a comprehensive synthesis of prior research and clearly situate the present findings within the broader research context. The corresponding revisions have been fully incorporated into the Discussion section of the revised manuscript, and we believe this adequately addresses the reviewer’s comment.

**Reviewer 2**

Comments 1: This research is interesting, however, the presentation is a bit confusing. I suggest that the authors reformate it in a more systematic ways. So, this research contains two stages of research.The first one is the development and validation of the medication adherence management attitude and behaviour scale and the second one is using that scale to measure factors influencing it (psychological contract and job burn out).

Response 1: We thank you for your positive evaluation of this study and for your valuable suggestions. In response, we have systematically revised the Methods and Results sections by explicitly structuring the study into two phases. The first phase focused on the development and psychometric validation of a scale assessing hospital pharmacists’ attitudes and behaviors toward medication adherence management. The second phase applied the validated scale to examine factors influencing pharmacists’ participation in medication adherence management.

To enhance clarity and logical coherence, we have added the following statement to the Methods section: “This study was conducted in two phases. In the first phase, a scale measuring hospital pharmacists’ attitudes and behaviors toward medication adherence management was developed and psychometrically validated. In the second phase, the validated scale was applied to investigate factors influencing pharmacists’ participation in medication adherence management” (lines 87–89). Following this revision, both the Methods and Results sections are now presented according to these two phases, resulting in a clearer and more structured presentation of the study design. Importantly, these revisions did not involve any substantive changes to the study content or data analyses.

Comments 2: The authors should systematically write it as two stages in the methods and explained it clearly the methods used for each stages. The combination of this two stages of research has make presentation of the results become too much with all 12 tables, with some of them are very big tables. This was not effective for an article. This has also makes the explanation about the important variables measured and other important part of methods were missing. One example include there is no information about the instrument used for psychological contract and job burn out, and what it is actually about.

Response 2: We thank you for your valuable comments on the manuscript structure and presentation of the methods. In response to your suggestions, we have systematically revised the Methods and Results sections by explicitly dividing the study into two phases. The first phase involved the development and psychometric validation of a scale assessing pharmacists’ attitudes and behaviors toward medication adherence management. The second phase applied the validated scale to examine factors influencing pharmacists’ participation in medication adherence management. To improve clarity and logical flow, we added the following statement to the Methods section: “This study was conducted in two phases. In the first phase, a scale measuring hospital pharmacists’ attitudes and behaviors toward medication adherence management was developed and psychometrically validated. In the second phase, the validated scale was applied to investigate factors influencing pharmacists’ participation in medication adherence management” (lines 87–89). Following this revision, both the Methods and Results sections are now organized by study phase, resulting in a clearer and more structured presentation, while the study content and data analyses remain unchanged.

With regard to your suggestion to reduce the number of tables, we sincerely appreciate and fully understand the reviewer’s concern. However, each table included in the manuscript serves a distinct and necessary purpose, specifically presenting: (1) the results of the Delphi expert consultation, (2) the psychometric properties of the developed scale, (3) the analysis of the relationship between pharmacists’ psychological contract and medication adherence management, and (4) the mediation analysis examining the role of job burnout. Each table corresponds to a different stage of data processing or statistical analysis, and merging or removing tables may compromise the completeness and clarity of the reported results. Therefore, to ensure transparency and scientific rigor, we respectfully request that all tables be retained.

In addition, we have added a new subsection entitled “Survey Instrument” to the Methods section (lines 129–144), which provides a detailed description of the instruments used in the study, including the Hospital Pharmacists’ Psychological Contract Scale and the Job Burnout Scale. This subsection also clarifies the content and scoring methods of each instrument, addressing previously missing but essential methodological information.

Comments 3: The authors has mentioned about the theories on the psychological contract and job burnout with work outcomes. But has not been strong enough why part of the work outcomes is their attitude and behaviour in medication adherence management and not the other pharmacist job? The authors need to provide the context and the problem for this for example whether this job is a mandatory task for pharmacist in hospitals in the study location, and what the problem about this specific task.

Response 3: We thank you for your valuable comments. In the current healthcare system in China, medication adherence management for patients is generally not a mandatory component of pharmacists’ formal job responsibilities, but rather an additional, proactive professional service. The core duties of most hospital pharmacists remain centered on prescription review, dispensing, medication consultation, and drug management, whereas adherence management activities—such as medication use education, follow-up monitoring, and interventions for inappropriate medication use—have not yet been explicitly incorporated into routine job descriptions or performance evaluation systems.

Accordingly, this study aims to examine the relationship between pharmacists’ perceptions of the psychological contract and their willingness to voluntarily invest time and effort in medication adherence management, thereby elucidating the motivating role of psychological contracts in shaping pharmacists’ proactive behaviors. This focus contributes to a better understanding of how psychological contracts influence pharmacists’ engagement in non-mandatory yet critical professional service activities. In response to the reviewer’s suggestion, we have added a detailed description of the study context and problem statement in the Introduction section of the manuscript (lines 48–61).

We sincerely appreciate the constructive feedbacks from the editor and reviewers, which have greatly helped us improve our manuscript. We hope that the revised version meets the journal’s standards and look forward to your kind evaluation.

Best regards,

Fushan Tang
