# Supplementary material for: The impact of hospital pharmacists’ psychological contracts on medication adherence management: The mediating role of job burnout
Source: PLoS One. 2026 Apr 29;21(4):e0347880. doi: 10.1371/journal.pone.0347880 (PMC13127971; doi:10.1371/journal.pone.0347880)
Supplement: Attachment — Submitted filename: Response to Reviewers-R3.docx [file pone.0347880.s005.docx]

***Fushan Tang, Professor, Ph.D***

Department of Clinical Pharmacy, Key Laboratory of Basic Pharmacology of Guizhou Province and School of Pharmacy, Zunyi Medical University, Zunyi 563006, China.

Email：fstang@vip.163.com; fstang@zmc.edu.cn.

*Tel*: *86851 28642337, Fax: 86851 28642334*

1 April 2026

Dear Editors,

Thank you very much for your letter dated December 8, 2025, informing us about the revisions required for our manuscript titled “The impact of hospital pharmacists' psychological contracts on medication adherence management: the mediating role of job burnout” (Manuscript ID: PONE-D-25-16578). We sincerely appreciate the valuable guidance, comments, and suggestions provided by the reviewers.

We have carefully reviewed all the comments and revised the manuscript accordingly. All modifications have been highlighted in the manuscript for easy identification. Additionally, we have provided detailed point-by-point responses to the reviewers' comments below.

**Reviewer #4**

Comments 1: The manuscript addresses an important topic related to pharmacists’ engagement in medication adherence management; however, the introduction would benefit from a clearer articulation of the research gap and the practical relevance of studying psychological contracts specifically in relation to adherence management rather than other pharmacy services.

Response 1: Thank you for your valuable comment. Regarding the concern about the research gap and the rationale for focusing on medication adherence management rather than other pharmacy services, we have provided a structured explanation in the Introduction and further clarified and strengthened it in the revised manuscript.

First, we explicitly state in the Introduction that, within the current healthcare system in China, medication adherence management is generally not part of the formal job responsibilities of most pharmacists, but rather represents an additional, proactive professional service (see lines 49–63). Compared with traditional pharmacy services, such as prescription review and dispensing, adherence management is characterized as “non-mandatory but essential,” and its implementation relies heavily on pharmacists’ individual initiative.

Second, based on psychological contract theory, employees’ perceptions of the fulfillment of organizational obligations are known to influence their work motivation. Since adherence management is not strictly driven by formal job requirements but depends largely on voluntary effort, it is particularly important to explore the underlying psychological mechanisms. Accordingly, this study proposes that pharmacists’ psychological contract perceptions may influence their willingness to invest additional time and effort in adherence management, thereby establishing a theoretical link between psychological contract and this type of proactive service behavior.

Furthermore, drawing on social exchange theory and conservation of resources theory, we clarify the theoretical pathway through which psychological contract affects work behavior via job burnout (see lines 64–73). Prior research has also highlighted that healthcare professionals’ work engagement is closely associated with their performance in medication adherence management.

Therefore, this study focuses on medication adherence management as a specific context to examine how psychological contract influences pharmacists’ participation in “non-mandatory but critical” professional services. This approach helps address a gap in the literature, which has predominantly focused on general job performance while paying limited attention to such discretionary service behaviors. We believe that the revised Introduction now clearly articulates both the research gap and the practical relevance of the study.

Comments 2: The study combines two distinct research stages (scale development and factor analysis) within a single manuscript, which makes the methodological presentation difficult to follow. The methods section should be reorganized to clearly separate the scale development phase from the analytical phase.

Response 2: Thank you for your valuable comment. In response to your suggestion to clearly separate the two research stages in the Methods section, we have made systematic revisions to the manuscript structure in the previous round.

Specifically, we have explicitly stated in the Methods section that the study was conducted in two phases, with the following clarification added: “This study was conducted in two phases. In the first phase, a scale measuring hospital pharmacists’ attitudes and behaviors toward medication adherence management was developed and psychometrically validated. In the second phase, the validated scale was applied to investigate factors influencing pharmacists’ participation in medication adherence management.”

In addition, both the Methods and Results sections have been reorganized and presented according to these two phases, ensuring a clear structural distinction between the scale development process and the subsequent empirical analysis, and improving the overall coherence and clarity of the manuscript.

We believe that these revisions have substantially enhanced the logical flow and readability of the methodological presentation, and clearly reflect the design and implementation of the two research phases.

Comments 3:The manuscript includes a very large number of tables, several of which present extensive item-level information that may not be necessary in the main text. Consider moving detailed scale-development tables to supplementary material to improve readability.

Response 3: Thank you for your valuable suggestion. In response to your concern regarding the large number of tables and the inclusion of extensive item-level details, we have revised the manuscript accordingly.

Specifically, detailed item-level information and related tables from the scale development process have been moved to the Supplementary Material (see Supplementary Material file). Only the key result tables that are directly relevant to the main findings have been retained in the main text, in order to highlight the core results and improve the overall readability and structural clarity of the manuscript.

We believe that these revisions effectively address the reviewer’s concern regarding table presentation, while maintaining the completeness and transparency of the study.

Comments 4: More detailed information about the measurement instruments used for psychological contract and job burnout should be included earlier in the methods section, including their original sources, validity, and reliability in previous studies.

Response 4:Thank you for your valuable comment. In response to your suggestion, we have further supplemented the validity and reliability information of the measurement instruments in the Methods section.

Specifically, in the “Survey Instrument” subsection, we have added the Kaiser–Meyer–Olkin (KMO) values and Bartlett’s test of sphericity for the Hospital Pharmacists’ Psychological Contract Scale. The KMO values for the three subscales were 0.957, 0.930, and 0.917, respectively (all > 0.6), and Bartlett’s test was statistically significant (p < 0.001), indicating good construct validity. In addition, Cronbach’s α coefficients and split-half reliability coefficients were all greater than 0.6, demonstrating acceptable reliability.

For the Maslach Burnout Inventory, we have supplemented the internal consistency coefficients (Cronbach’s α) for its three dimensions, which were 0.89, 0.79, and 0.87, respectively, further supporting the reliability of the scale.

These additions provide more comprehensive information on the sources, structural characteristics, and psychometric properties of the instruments, and enhance the rigor and completeness of the Methods section.

We sincerely appreciate the constructive feedbacks from the editor and reviewers, which have greatly helped us improve our manuscript. We hope that the revised version meets the journal’s standards and look forward to your kind evaluation.

Best regards,

Fushan Tang
